# Advancing Equity in the Farm Bill: Opportunities for the Gus Schumacher Nutrition Incentive Program (GusNIP)

**DOI:** 10.3390/nu15234863

**Published:** 2023-11-21

**Authors:** Sara John, Blanca Melendrez, Kirsten Leng, Amy Nelms, Hilary Seligman, James Krieger

**Affiliations:** 1 Center for Science in the Public Interest, Washington, DC 20005, USA; anelms@cspinet.org; 2 Center for Community Health, University of California San Diego, La Jolla, CA 92037, USA; bmelendrez@ucsd.edu; 3 Healthy Food America, Seattle, WA 98122, USA; kirsten@kirstenlengconsulting.com (K.L.); or jwkrieg@uw.edu (J.K.); 4 Division of General Internal Medicine, University of California San Francisco, San Francisco, CA 94143, USA; hilary.seligman@ucsf.edu; 5 School of Public Health, University of Washington, Seattle, WA 98195, USA

**Keywords:** nutrition incentives, fruit and vegetable intake, food security, nutrition security, health equity, Gus Schumacher Nutrition Incentive Program (GusNIP), Supplemental Nutrition Assistance Program (SNAP), farm bill, food policy

## Abstract

The Gus Schumacher Nutrition Incentive Program (GusNIP) is a federally funded grant program that provides nutrition incentives—subsidies for purchasing fruits and vegetables (FV)—to Supplemental Nutrition Assistance Program (SNAP) participants. GusNIP currently advances nutrition equity by improving FV access for people with low incomes, yet inequities exist within GusNIP. We sought to identify inequities in GusNIP at the community, organization, partner, and individual levels and develop recommendations for farm bill provisions to make the program more equitable. In Spring 2021, a group of nutrition incentive experts (*n* = 11) from across the country convened to discuss opportunities to enhance equity in GusNIP. The iterative recommendation development process included feedback from key stakeholders (*n* = 15) and focus group participants with GusNIP lived experience (*n* = 12). Eleven recommendations to advance equity in GusNIP in the farm bill emerged across six categories: (1) increase total GusNIP funding, (2) increase funding and support to lower-resourced organizations and impacted communities, (3) eliminate the match requirement, (4) support statewide expansion, (5) expand and diversify retailer participation, and (6) expand program marketing. Including these recommendations in the upcoming and future farm bills would equitably expand GusNIP for SNAP participants, program grantees, and communities across the country.

## 1. Introduction

The epidemic of diet-related chronic diseases continues to grow in the United States. Type 2 diabetes, hypertension, obesity, and cardiovascular disease affect large portions of the population [1,2,3,4]. These chronic conditions disproportionately affect Black, Latine, and Indigenous Americans and people with lower incomes, leading to large health inequities [1,2,3,4]. For example, Black and Latine individuals experience higher rates of diabetes and associated complications, and Hispanic and Indigenous American populations show a higher prevalence of hypertension compared to non-Hispanic Whites [5]. These inequities are driven by structural racism and other social determinants of health including poverty, unemployment, racism, and neighborhood conditions [6,7]. Table 1 defines key principles and concepts used throughout the paper related to the Gus Schumacher Nutrition Incentive Program (GusNIP), a federally funded grant program that strives to address nutrition and health inequities by making fruits and vegetables (FVs) more accessible through financial FV incentives for people with lower incomes.

The food environment, including access to healthy and nutritious foods such FVs, is an important social determinant of health, inequities, and chronic disease prevalence. The 2020 Dietary Guidelines for Americans emphasize the prominent role of FVs in a healthy diet [19]. Eating FVs is associated with decreased risk of cardiovascular disease, type 2 diabetes, and some types of cancer [20,21]. Despite this, only one in ten Americans consume recommended amounts of FVs [22], which is unsurprising given the complexities of accessing and maintaining a healthy diet in the U.S. food environment [23,24,25,26]. While many environmental barriers to a healthy diet exist, the lack of affordability of foods that are part of a healthy diet is a primary barrier for people with limited financial resources [27]. This contributes to lower FV intake among people with low incomes relative to those with more resources [28,29]. Additionally, FVs are often more expensive than less healthful and extensively marketed foods such as ultra-processed products [30]. As a result, people from food-insecure households are less likely to consume enough FVs because they are unable to afford them [31,32,33]. The impact of food insecurity is further enhanced within racial-ethnic minority groups, with Black and Latine individuals disproportionately affected by food insecurity along with those living in households with incomes below the federal poverty level (FPL) [34]. Recent studies indicate Black and Latine persons experience food insecurity at rates of 21.2% and 16.2%, respectively, nationwide, compared to the national average of 11.2% [35]. In addition, high-quality FVs are not accessible in many impacted communities [36], making access to FVs a critical health equity issue in communities with limited resources across the country. These disparities are driven by historical and structural factors and racial and social inequities including poverty, unemployment, discrimination, and racism [35,37] that limit access to affordable healthy foods including FVs [6,7], pointing to a need to address such factors that produce and exacerbate social inequities in order to effectively address healthy food access amongst underserved and racial-ethnic minority communities.

Nutrition incentives (NIs) help people purchase and consume FVs by providing financial subsidies, rebates, or discounts for FVs, thus making them more affordable. A robust body of evidence shows that NIs increase purchase and consumption of FVs [38,39,40,41,42,43,44,45,46,47,48,49], improve food security [44,49,50], and provide economic benefits to impacted communities [44,51,52]. Microsimulation and econometric models suggest that NIs lead to improved health outcomes and are cost effective [53,54].

Given the need to improve access to FVs for people with low incomes and the effectiveness of NIs, Congress established GusNIP and its predecessors, the Healthy Incentives Pilot (HIP) and the Food Insecurity Nutrition Incentive (FINI) programs [55,56,57]. GusNIP was authorized in the 2018 Farm Bill, the omnibus, multi-year legislation that governs U.S. agriculture and food programs, including the Supplemental Nutrition Assistance Program (SNAP), the largest domestic food assistance program [56]. The legislation authorized a five-year $250 million budget administered by the U.S. Department of Agriculture (USDA) [56]. In 2021, USDA’s GusNIP COVID Relief and Response initiative awarded an additional $69 million to active GusNIP and FINI grantees to “address critical food and nutrition security needs of low-income communities, enhance the resilience of food and healthcare systems impacted by the pandemic, and maximize funds reaching participants” [58].

GusNIP has two components: the NI program, which is the focus of this paper, and the produce prescription program, which we do not discuss [12]. The NI program awards approximately $35 million annually to state and local organizations to implement NI programs in their communities [59]. Pilot, standard, and large-scale project grants are available through a competitive application process to non-profit and government agencies throughout the United States. The provision of dollar-for-dollar matching funds is required for funded grantees [60]. As of 2023, there are 76 active NI grants [61].

Programs funded through GusNIP provide NIs for the purchase of FVs to people participating in SNAP, which can be utilized at participating SNAP-authorized retailers to purchase fresh, canned, dried, and/or frozen FVs which do not contain added sugars, fats/oils, or salt [59]. For each SNAP dollar spent on eligible FVs, participants receive a matching amount of NIs. Program designs vary across grantees with respect to the types of FVs included, cash value of incentives, incentive mechanism (e.g., tokens, paper vouchers, Electronic Benefits Transfer (EBT) cards), populations served, and retailer types [41,62]. Participating retailers include farmers markets, co-ops, supermarkets, grocery stores, and corner stores [62].

GusNIP is by design focused on reducing nutrition disparities because it provides benefits to people with low incomes who are most impacted by nutrition inequities. GusNIP NIs are distributed to SNAP participants, who generally have household incomes at or below 130% FPL and who are predominantly non-white [63]. An estimated 54% of recent GusNIP participants are food insecure [44]. Since its inception, GusNIP has functioned as an important and effective tool for increasing FV affordability and intake and food security in communities with limited resources. In fact, GusNIP provided NIs in communities where, on average, 14.1% of the community members have incomes below the FPL, compared to 11.4% nationally [44]. GusNIP also benefits these communities economically [44].

While acknowledging GusNIP’s contributions to advancing nutrition equity, it is also important to recognize the inequities that exist within GusNIP at the community, organization, partner, and individual levels. Successful GusNIP grantees are often larger, well-established, white-led organizations that have the capacity to generate the required match dollars, prepare competitive grant applications, and administer program activities [64]. Funded GusNIP projects are unevenly distributed across the country [64]. For example, nearly half (45%) of all GusNIP funds awarded between 2019 and 2022 were allocated to three states [64]. No funds for NI programs have been awarded to tribal agencies [64]. Projects in under-represented regions and impacted communities are more likely to be standard or pilot projects with lower funding relative to the large-scale projects found elsewhere [64]. Because Black, Latine, and Indigenous people are generally under-represented among farmers [65] and food retail store owners [66], they are also likely to be under-represented among FV suppliers who participate in GusNIP-funded programs. At the individual level, there is a greater prevalence of white participants in GusNIP (52%) relative to the general SNAP population (37%) [44,63]. The nearly trillion-dollar farm bill is renewed every five years and is the largest legislative opportunity to pass key nutrition and agriculture policies, including GusNIP [9]. The upcoming farm bill could make policy changes and authorize funds to develop a more comprehensive approach to equity within GusNIP. The recommendations proposed in this paper are a timely path for moving forward, enabling GusNIP to more effectively address the root causes of food and nutrition insecurity and advance equity in alignment with the needs of communities with limited resources nationwide.

## 2. Materials and Methods

The GusNIP equity recommendations discussed in this paper were developed between March 2021 and October 2022 with the intention of informing the renewal of the farm bill in 2023. In Spring 2021, a group of NI experts from across the country came together to discuss approaches and opportunities to enhance equity in GusNIP through the farm bill. Our workgroup consisted of people (*n* = 11) who work in national, non-profit organizations, academia, agriculture outreach centers, and community health improvement. Many were female, most were white, and all were food secure. These positions influence our knowledge, experience, and views.

We started with an initial set of questions to frame our discussions:

- How do we build capacity and infrastructure to get a more diverse group of grantees and retailers offering NIs? Are there changes needed to the local match requirements to further support equity? How else can barriers to applicants and retailers be addressed?
- How do we expand participant reach to increase the diversity of those who receive NIs? What is known about the demographics of NI participants? How does this compare to SNAP demographics?
- How do we build community and participant engagement into the NI program planning and implementation process?
- What else could be done to make GusNIP more equitable?

We hosted an iterative discussion process via a series of six virtual meetings and follow-up email correspondence to discuss these questions, identify program inequities, and develop an initial set of recommendations. We cross-walked these initial recommendations with those that emerged from the 2021 GusNIP Theory of Change (TOC) development process. Leng et al. provides a detailed description of the GusNIP NI TOC methodology and findings [67]. In summary, the process engaged GusNIP participants, practitioners, and partners to develop a TOC to describe how and why GusNIP NI projects work. During the process, several themes emerged describing opportunities for how GusNIP could be more centered in equity and community. Opportunities from the TOC process not yet captured were added to the initial set of recommendations.

Six members of the workgroup volunteered to be a part of a writing group. This group researched and fleshed out each of the initial recommendations. The writing group shared these initial recommendations via email with the other workgroup members as well as additional key stakeholder reviewers (*n* = 10) including food retailer associations, academic experts, tribal representatives, and successful and unsuccessful GusNIP grant applicants to secure their feedback (see Table 2).

Additionally, we hosted a focus group in San Diego, CA, to ensure the perspectives of people with lived experience using GusNIP NIs were included in the recommendations. Participants (*n* = 12) were either participating in the University of California San Diego (UCSD) ¡Mas Fresco! program, a current GusNIP NI program in San Diego County, or recruited from a multi-racial, multi-ethnic Community Council comprised of San Diego County residents advocating for healthier communities under the San Diego County Childhood Obesity Initiative, facilitated through the UCSD Center for Community Health. Those community members who took part in the focus group all consented to participate and to provide their names in association with the feedback that emerged (see Acknowledgements). The 1.5 h focus group was conducted over Zoom and facilitated by a workgroup member in Spanish to accommodate the linguistic needs of participants. The focus group was recorded with participant permission. A Spanish–English translator provided real-time translation for recording purposes, and audio recordings were transcribed using a professional transcription service. Participants received a $50 gift card incentive.

Qualitative analysis of focus group transcripts and summaries was conducted using rapid qualitative and thematic analysis methods. One workgroup member reviewed the focus group transcript and summarized the data for each recommendation in Microsoft Word version 2310. She then compiled data from the key stakeholder reviewers and focus group into a revised draft of recommendations that noted agreement with and challenges, and additions to the initial recommendations. The writing group used this information to inform a consensus-based process to incorporate modifications and suggestions into the initial recommendations. Prior to finalization, the initial recommendations were shared back with the same community members who participated in the above-described focus group to ensure agreement with the proposed recommendations; no points of disagreement were reported. This was conducted through a one-hour virtual meeting facilitated in Spanish over Zoom by the same workgroup member who facilitated the original focus group, and the community members who participated all agreed with the recommendations. Finally, the larger workgroup used a ranked-voting system to prioritize recommendations for the upcoming farm bill. This paper summarizes the final recommendations from this analysis.

## 3. Results

We first summarize GusNIP features that contribute to inequities, and then describe opportunities and make recommendations for advancing equity in GusNIP in the farm bill.

### 3.1. Features of GusNIP Contributing to Program Inequities

Our discussions identified seven aspects of GusNIP that contribute to program inequities at the community, organization, partner, and individual levels (see Table 3). They include financial, organizational capacity, technical, and community engagement barriers to full and equitable participation.

### 3.2. Recommendations to Advance Equity in GusNIP through the Farm Bill

Eleven recommendations to advance equity in GusNIP in the farm bill emerged across six categories: (1) increase total funding, (2) increase funding and support to lower-resourced organizations and impacted communities, (3) eliminate the match requirement, (4) support statewide expansion, (5) expand and diversify retailer participation, and (6) expand program marketing (Table 4). These opportunities and corresponding recommendations are described in more detail in the sections that follow.

#### 3.2.1. Increase Total GusNIP Funding

Only a small fraction of people enrolled in SNAP are currently able to access incentives. In the past year, approximately $40 million worth of GusNIP NIs were redeemed annually to reach 146,000 participants monthly [44]; but, if all 22 million households participating in SNAP received $20 of incentives per month, the annual cost of incentives alone would be $5.2 billion [68]. Projects report that available funds for incentives are inadequate to meet demand [69]. For example, funds may be exhausted before the end of the year, or enrollment may be closed to new participants (who may be placed on waiting lists instead). Additional funds are needed for incentives and program implementation to expand the reach of GusNIP.

Many of the subsequent equity-centered recommendations for increasing equity within GusNIP will also require additional funding, especially to lower-resourced organizations and impacted communities. The 2018 Farm Bill authorized $250 million for GusNIP over five years [56]. We recommend the farm bill increase GusNIP appropriations to $7 billion over ten years. While this allocation would still not allow GusNIP to reach all SNAP participants, it would represent an initial step towards expanding reach and improving equity. Strategically deployed, increased appropriations for GusNIP in the next farm bill will make GusNIP more equitable and accelerate equity in food and nutrition security and dietary intake.

#### 3.2.2. Increase Funding and Support to Lower-Resourced Organizations and Impacted Communities

Inequities in the funding of GusNIP sites are driven in part by the barriers faced by impacted communities when applying for GusNIP grants. Impacted communities often have less capacity to prepare applications. They may propose projects that use non-traditional approaches for program implementation which may not score as well as typical GusNIP projects, for example, emphasizing deeper and more holistic engagement with community members over maximizing the number of participants receiving incentives.

Grant application reviews may reward proposals with certain writing and presentation styles, favor applications based on technical merit, and not sufficiently recognize community needs and values. The farm bill should increase funding for small-scale pilot projects to allow lower-resourced organizations in impacted communities to develop the experience and skills needed to develop proposals for larger projects and implement them, while not competing with higher-capacity and more experienced organizations. Small-scale pilot projects should not require any grantee funds for matching.

GusNIP currently funds the GusNIP NTAE to support grantees. While the GusNIP NTAE offers feedback to unsuccessful applicants about why they did not receive funding and ways to improve future applications, proactive support prior to application submission has traditionally been minimal and limited to a one-hour consultation with the GusNIP NTAE [59]. The GusNIP NTAE has recently started offering Capacity Building and Innovation Fund Application Support Grants aimed at supporting capacity building for organizations who have not yet applied for or successfully received a GusNIP award [70]. With additional funding and authorization to expand the scope and reach of these types of support grants and services, the GusNIP NTAE should increase technical assistance to lower-resourced organizations in impacted communities. Assistance should support the development and implementation of successful community-centered projects, leading to greater diversity among grantees. The upcoming farm bill should enable this expansion of technical support with increased funding to the GusNIP NTAE. 

We recommend that GusNIP expand planning grants to allow lower-resourced organizations from impacted communities to build capacity and implement community-based application development, needs assessment, and project planning. The work conducted during the planning period would be used to prepare a strong and community-centered application for GusNIP project funding.

#### 3.2.3. Eliminate the Match Requirement

GusNIP NI grant applicants must match federal funds dollar-for-dollar (50% of program costs funded using GusNIP funds and 50% using applicant sources) [59]. Matching sources may include cash contributions from public and private sector funders and certain types of in-kind contributions [59]. Federal funds cannot be used as match contributions except in the case of tribal agencies [61]. Meeting the match requirement has become a significant barrier for some applicants, particularly those from lower-resourced organizations with limited existing funding [69].

The 2018 Farm Bill defined the matching requirements for USDA National Institute of Food and Agriculture (NIFA) competitive grants, including GusNIP [56]. Notably, almost no other NIFA grant programs require a match, and those that do have match rates are all lower than the dollar-for-dollar match required for GusNIP. Others waive the match requirement if the USDA Secretary deems this is “necessary to effectively reach an underserved area or population”, a condition that is met given the current distribution of GusNIP grantees. We recommend the next farm bill eliminate the requirement for grantee-generated matching funds. If there are inadequate funds to drop the match requirement, a significant reduction in the match requirement for all grantees would still reduce barriers to participation. If a universal match requirement decrease is not feasible, the farm bill could eliminate or reduce match requirements for lower-resourced organizations in impacted communities.

Additionally, the farm bill could expand what qualifies as a match and how a match is calculated to reduce barriers. Federal dollars or volunteer time could be used as matching funds. Another strategy might be limiting the match requirement to the portion of grant funds reserved for program operation expenses, with no matching funds required for the portion of grant funds reserved for the NIs.

#### 3.2.4. Support Statewide Expansion

Statewide SNAP incentive programs are a critical intermediate step towards a national NI program available to all SNAP participants, yet they are not currently funded under GusNIP. The next farm bill should include a new funding category to support statewide expansion of GusNIP to provide key insights for national expansion and universal NI access for people that participate in SNAP. Farm bill funding should also support essential state administrative work and statewide EBT integration of NIs, which may lead to expanded participation and redemption. EBT integration refers to an NI program where the benefits are placed directly on the SNAP EBT card for shoppers to seamlessly earn and redeem NIs as they spend their SNAP benefits at SNAP-authorized retailers.

Currently, NIs are distributed using electronic methods (e.g., store loyalty cards, automatic discounts) and physical methods (e.g., paper coupons, tokens) [44]. Approximately 72% of GusNIP-participating retailers use physical incentives, like tokens and paper vouchers [44]. Electronic methods for NI delivery such as EBT card integration could expand access to NIs, simplify transactions, reduce stigma, improve redemption with elimination of lost vouchers, and streamline program operations [69]. Integrating NIs into the broader SNAP system lays the groundwork for a national SNAP incentive program that can be more widely accessible for shoppers utilizing SNAP. However, it is also important to acknowledge that electronic methods may also drive NI use to retailers with capacity for advanced point-of-sale (POS) systems, sometimes lacking in community stores. This reinforces the need to provide technical assistance to these retailers to implement the appropriate technology for electronic NI processing. State integration of NIs with the existing SNAP EBT card is an alternative electronic incentive distribution solution that is already underway in states like Massachusetts, Washington, and California. Although a significant resource investment, funding state programs to implement this integration represents an important opportunity to increase participation and redemption. USDA has already made some investment in EBT integrated models with a cooperative agreement RFA released in early 2023 [71]. However, ten states are already pursuing statewide expansion, and as more states move towards operating truly statewide programs, a more consistent funding stream is necessary [72].

#### 3.2.5. Expand and Diversify Retailer Participation

Currently, 2928 retailers participate in GusNIP [44], representing about 1% of all SNAP-participating retailers [73]. Most (84%) GusNIP-participating retailers are in urban areas [44]. GusNIP aims to engage multiple types of food retailers. Among GusNIP NI retailers, 63% are farm direct sites (e.g., farmers markets, farm stands, mobile markets) and 37% are brick-and-mortar sites (e.g., grocery stores, supermarkets, convenience stores) [44]. Despite being a smaller percentage of all participating retailers, brick-and-mortar sites redeem a large share of NIs (54%) [44]. In previous years, small grocery and convenience stores have only accounted for a small fraction (8%) of brick-and-mortar sites [62].

Increasing the participation of community-owned food retailers—independent food retailers including small grocery stores, convenience stores, and food co-ops that are more likely to be owned by Black, Latine, or Indigenous people who reflect the community—would increase equity in GusNIP. These retailers play an outsized role in impacted communities where supermarkets are less likely to locate [74], and long-standing small businesses are more likely to be owned by Black, Latine, or Asian community members [66]. Their locations within communities may improve geographic accessibility for some shoppers—particularly people impacted by social determinants of health, with physical limitations, and/or with limited economic and time resources [75,76,77]. These smaller food retailers may also serve rural areas where current GusNIP retailer participation is limited [44]. In addition, these stores may be more likely to stock culturally relevant foods and may be retailers of choice for some NI participants. Revenue generated from the redemption of NIs supports these businesses directly, and purchases of other products by NI participants further increases sales. Offering a greater variety of food retailers increases participant choice of where to redeem NIs. These advantages can outweigh the potential disadvantages associated with including these stores, such as potentially higher prices, smaller variety of available FVs, and lack of access to technology needed for electronic NI issuance and redemption.

Several barriers limit the participation of community-owned food retailers. Enrolling multiple community-owned retailers as NI redemption sites requires more effort by grantees, relative to working with a single large corporate retailer that operates several stores in a community. Grantees may lack the capacity (both time and technical skills) for recruiting and supporting small retailers, particularly because these retailers may need more assistance from grantees than larger stores that have technology in place to support redemption processes. For example, the most streamlined system for NI redemption is a POS system that automatically triggers a discount or instantly places the additional benefit on FVs when a customer uses a SNAP EBT card. Yet POS systems are expensive and difficult for local store owners to set up without technical support and resources. Thus, we also recommend that the upcoming farm bill provide community-owned food retailers with technical and financial support for implementing electronic NI issuance and redemption technology.

#### 3.2.6. Expand Program Marketing

Awareness of NIs is low among people who participate in SNAP. For example, only 31% of those living near a retailer participating in FINI were aware of the program [78]. Currently, USDA restricts how grant funds for project marketing can be used and does not provide guidance on evidence-based promotional strategies [59].

Nearly all retailers that offer NIs utilize on-site marketing and signage (89%), but online advertisements are much less commonly utilized (43%) [44]. Digital strategies could be a cost-effective approach to expanding promotion [79]. Advertising through social media and apps is a promising and scalable approach given the near ubiquity of smart phone ownership and social media usage by people of all races, ethnicities, and income levels [80,81]. Digital strategies could also include in-app advertising within EBT management apps, like Providers, and promotion of the Shop Simple with MyPlate app that already provides GusNIP locations and could be expanded to include program promotion and marketing [82]. Additionally, SNAP-Education (SNAP-Ed) funds could be used for digital marketing of NI programs [83].

Tailoring program promotions to diverse audiences would also be useful to reach a diverse SNAP participant audience. Across all GusNIP-participating retailers, 33% provide multilingual marketing promotions [44], and these efforts could be expanded, including greater availability of marketing materials in multiple languages, multilingual volunteers on site to explain programs, a multilingual customer hotline, and hiring community members fluent in the preferred languages of local SNAP participants as program and retail staff. Each of these strategies requires additional investment of time and resources. Therefore, we recommend the farm bill authorize funds for USDA to develop federally supported, national GusNIP promotions and offer supplemental funding to grantees for promotional activities tailored to their communities.

## 4. Discussion

The foundation for advancing equity in GusNIP is increased funding so more people using SNAP can benefit from NIs. A substantial increase in GusNIP appropriations in the next farm bill could fund more NIs and prioritize program expansion to more impacted communities. To expand NI availability in impacted communities that currently lack GusNIP projects, the farm bill should also increase financial and technical support to lower-resourced organizations throughout the project cycle from development to proposal submission to implementation to evaluation to sustainability planning. Eliminating the match requirement would also reduce a major barrier to GusNIP entry and allow greater participation of lower-resourced organizations. Additionally, the farm bill should support equitable GusNIP implementation through the diversification of retailer participation with the authorization of funding and technical assistance for community-based retailers that serve impacted communities.

The upcoming farm bill can also advance equity in GusNIP by making NIs more accessible. Allocating funding for national, digital promotions of NIs as well as support for community-specific marketing that is culturally and linguistically tailored to diverse audiences will increase the use of NIs. Ultimately, NIs should be available and accessible to all SNAP participants. Supporting the expansion of state projects and the integration of NIs into SNAP EBT systems will create a seamless participant and retailer experience, a critical intermediate step towards making NIs a core element of the SNAP program for all participants. The next farm bill should prioritize these steps. Key advocacy and stakeholder groups at the national and state level also support many of these recommendations [72].

Our recommendations also align with many of the practices of other federal and philanthropic grant programs that support the development and funding of grant applications from lower-resourced organizations and impacted communities. The CDC Communities Putting Prevention to Work program included a $10 million fund to support higher-capacity applicants willing to provide peer-to-peer mentorship to lower-resourced organizations [84]. The Robert Wood Johnson Foundation’s Evidence for Action program offers technical assistance services to applicants who propose research that is relevant to advancing racial equity, but whose projects do not meet all its criteria for rigor, actionability, or research team qualifications [85]. It offers select applicants the opportunity to receive project design consultation or matching services that facilitate partnerships between community-based organizations and experts for developing proposals [85]. Additionally, CDC REACH grants require recipients to engage an established community coalition to help develop projects and to use “community-specific best practices” to implement activities, monitor progress, and oversee communications with communities [86]. The coalition must have “priority population representatives” as well as a representative from a local community-based organization [86]. The Kresge Climate Change, Health and Equity Initiative requires applicants to be a part of a multi-disciplinary partnership and the lead applicant to be representative of the community [87]. Tribal organizations are encouraged to apply, and preference is given to organizations with Black, Latine, and Indigenous leadership and those that partner with underserved communities [87].

Elimination or reduction of the matching requirement would further align GusNIP with other federal programs that address nutrition, social determinants of health, and equity that require limited or no matching fund contributions. For example, the CDC REACH program, the CDC Communities Putting Prevention to Work initiative, and the Department of Housing and Urban Development Community Development Block Grants do not have matching requirements [84,86,88]. The federal matching rate for Medicaid is based on state per capita income. The lowest income states contribute 22–30% in match, while the highest income states contribute 50% [89]. Other legislation has more inclusive definitions of match. For example, the Surface Transportation Reauthorization Act of 2021 allowed a state’s Department of Transportation to use federal Highway Safety Improvement funds as match [90].

Our recommendations may have potential unintended consequences. First, there may be a trade-off between deepening equity in GusNIP program implementation and expanding program reach. For example, awarding additional operating funds to lower-resourced organizations and impacted communities would likely increase the diversity of funded projects, yet it may also reduce GusNIP program funds available for direct incentives. Providing preference to lower-resourced organizations and higher-need communities may increase GusNIP presence in communities experiencing the greatest inequities. However, funding higher-capacity organizations with existing large and efficient GusNIP projects may serve people with similar needs at a lower cost per participant and may therefore increase participation. Integrating NIs into state SNAP programs may advance equity by expanding the reach of GusNIP projects, making incentives more accessible to more people. However, if the project is administered by a state agency, this may reduce opportunities for people from impacted communities to play a role in project design and implementation. GusNIP expansion must include impacted communities’ voices.

Second, collecting the data necessary to qualify for designation as a lower-resourced organization or impacted community may place greater burdens on applicants from the very populations most impacted by inequities. Data requirements should be designed to be the least burdensome possible.

Third, increasing participation of community-owned food retailers may increase equity in retailer participation. However, most SNAP participants spend their benefits at supermarkets and super stores, making expansion among these retailers also important [91]. Additionally, if FV prices are higher in smaller stores, then the purchasing power of NIs is reduced.

We also recognize several limitations in our process for developing these recommendations. Our community input included the engagement of interested parties and facilitation of a focus group with community members currently utilizing GusNIP NIs. Future efforts should further center community voice through expanded efforts to gather and prioritize the perspectives of diverse community members and people who use GusNIP, community retailers, and community-based practitioners who implement NI programs. Increased community engagement and the integration of a community-based participatory approach to gathering continual feedback on GusNIP from communities impacted by health inequities could facilitate a deeper understanding of equity issues and solutions for GusNIP.

We note that communities differ, and the most equitable and effective approaches to NI implementation may vary across communities. Respect for the specific conditions, cultures, priorities, and goals of each community must be balanced with consideration of national program requirements and guidelines based on evidence and best practices. GusNIP implementation should balance support for evidence-based approaches while allowing opportunities for community-driven input and innovation. To address systemic inequalities including structural racism, impacted communities should have the opportunity to participate in project design and implementation. Thus, USDA must allow some degree of flexibility and autonomy in NI implementation while also adhering to national standards and considering evidence of what works.

The recommendations we offer intentionally focus on elements of the farm bill that could advance equity within GusNIP. During this process, we also developed recommendations for equitable expansion of GusNIP through USDA administrative action (see Table S1). In addition to these recommendations, we also recognize that there are upstream structural issues and barriers affecting equitable access to healthy foods for under-resourced communities, including the broader impacts of systemic racism and climate change amongst other issues. Future efforts to improve equitable access to healthy foods must incorporate community-driven strategies to address structural issues and root causes of nutrition insecurity in impacted communities.

Looking beyond the next farm bill, we envision a future where all SNAP participants can access NIs. A national SNAP incentive program could provide efficient, streamlined NIs that are integrated into the EBT system and available at all SNAP-authorized retailers. While universal access to NIs for all people using SNAP would be a major advance for food and nutrition security, this alone would not fully realize the original intent of GusNIP. Much like the farm bill itself, GusNIP goals include both the support of local agriculture and provision of nutrition assistance. At farmers’ markets, GusNIP subsidizes purchases of locally-produced FVs, thus increasing sales and supporting the local farmers while also improving the nutrition security of GusNIP participants who can now afford to buy more FVs. Does a state or national NI program where most NIs are redeemed at supermarket chains advance food and nutrition security goals to the detriment of local and regional food system goals? Does the continued focus of GusNIP expansion in farmers’ markets, where 0.02% of SNAP benefits are spent, prioritize local food systems over food and nutrition security? Universal access to NIs and support of local retailers, farmers, and community organizations must be balanced.

## 5. Conclusions

We envision an expanded GusNIP program centered in equity that provides NIs to many more people participating in SNAP and includes more diverse, under-resourced communities and organizations. An equity-centered and community-based GusNIP program will move us closer to being a nation in which all people are able to eat healthy food and enjoy good health.

We offer several recommendations to realize this vision:

- Increase total funding for the GusNIP program to increase the amount of NIs provided and the number of participants.
- Support greater participation of lower-resourced organizations and communities impacted by nutrition insecurity, diet-related diseases, and structural racism.
- Eliminate the requirement that grantees match federal grant award funds to remove a financial barrier of GusNIP participation.
- Support statewide expansion of GusNIP to simplify and streamline participation of people using SNAP and food retailers.
- Diversify the types of food retailers participating in GusNIP to increase representation of small and independent community-based retailers.
- Expand promotion and marketing of the GusNIP program and state and local projects to increase awareness and participation.

Moving forward, we encourage policymakers to incorporate the recommendations outlined here as part of the upcoming farm bill. We further urge policymakers, funders, and community advocates to center community needs and strategies that improve equity in future farm bills, administrative actions, and other policy and grant-making initiatives. As NIs grow through GusNIP and beyond, in the next farm bill and future farm bill cycles, equitable expansion should be prioritized.

## Figures and Tables

**Table 1:** Key principles and concepts related to the Gus Schumacher Nutrition Incentive Program (GusNIP).

Term	Definition
Community-owned food retailers	Independent food retailers with leadership that reflects the community, including small grocery stores, convenience stores, food co-ops, and farm direct sites.
Equity	The condition that would be achieved if one’s social status, including race, income and wealth, and place of residence no longer influenced how one fares [8].
Farm bill	The farm bill sets national agriculture, nutrition, conservation, and forestry policy and is passed by Congress every five years. It includes Title IV, the nutrition title that authorizes SNAP and other federal food assistance programs [9].
Food environment	The physical, social, economic, cultural, and political factors that impact the availability, accessibility, affordability, and quality of food within a community or region [10].
Food insecurity	A household-level economic and social condition of limited or uncertain access to adequate food [11].
Gus Schumacher Nutrition Incentive Program (GusNIP)	The Gus Schumacher Nutrition Incentive Program (GusNIP) awards organizations with competitive grants to conduct and evaluate projects that provide incentives for individuals with low incomes to increase their purchase of fruits and vegetables (FVs) and prescriptions for these foods. Since 2019, $270 million in funding has been distributed to 197 projects across the U.S. through GusNIP [12].
GusNIP Nutrition Incentive Program Training, Technical Assistance, Evaluation and Information Center (NTAE)	A coalition of partners awarded $8.5 million by the U.S. Department of Agriculture (USDA) in the 2019 fiscal year to provide support to nutrition incentive and produce prescription projects [13].
Impacted communities	Communities disproportionately impacted by above-average rates of poverty, food insecurity, unemployment, or diseases associated with poor nutrition.
Lower-resourced organizations	Organizations with lower-than-average access to funding, social networks, administrative infrastructure, and/or expertise in securing and implementing federally funded grants.
Match requirement	A portion of a project’s costs are not paid for by the grant and must be covered by the grantee. Match requirements are typically stated as a percentage of the total amount of funds awarded [14].
Nutrition incentives (NIs)	Financial incentives (subsidies) for the purchase of FVs.
Nutrition-related disease	Diseases associated with poor dietary patterns including heart disease, type 2 diabetes, and obesity [15].
Nutrition security	Consistent access to safe, healthy, affordable foods essential to optimal health and well-being [16].
Social determinants of health	The conditions in the environments where people are born, live, learn, work, play, worship, and age that affect a wide range of health, functioning, and quality-of-life outcomes and risks, including economic stability, education access and quality, healthcare access and quality, neighborhood and built environment, and social and community context [17].
Structural racism	The totality of ways in which societies foster racial discrimination through mutually reinforcing systems of housing, education, employment, earnings, benefits, credit, media, health care and criminal justice that reinforce discriminatory beliefs, values, and distribution of resources [18].

**Table 2:** GusNIP equity recommendation contributors and roles.

Type of Contributor (*n*)	Role
Workgroup member (11)	People representing non-profit organizations, agriculture outreach centers, academia, and community health improvement organizations. Brought nutrition incentive (NI) expertise. Generated and prioritized initial set of recommendations.
Writing group member (6)	Subgroup of workgroup described above. Drafted and finalized recommendations.
Key stakeholder reviewer (10)	Food retailer associations, academic experts, tribal representatives, and successful and unsuccessful GusNIP grant applicants. Brought NI administration and implementation experience as well as academic and community perspectives regarding NI impacts and best practices. Reviewed initial recommendations and provided feedback.
Focus group participant (12)	Community members who had used NIs. Brought community perspective and lived experiences. Reviewed initial recommendations and provided feedback.

**Table 3:** Features of GusNIP contributing to program inequities.

Level	Program Feature	Impact on Equity
Community ^1^/ Organization ^2^	Grantees must match federal funds dollar-for-dollar with local resources.	Creates financial barrier to GusNIP participation, especially for lower-resourced organizations and impacted communities.
	Organizations located in impacted communities frequently have lower resources to prepare successful grant applications.	Creates resource barrier to GusNIP application for lower-resourced organizations and impacted communities.
	The application review process emphasizes the technical merit of the application over community need.	Fails to prioritize impacted communities where need is greatest.
	Grants often do not provide sufficient support for operating costs.	Disadvantages smaller, lower-resourced organizations that lack existing operational capacity.
	Community member participation and leadership in local project design and implementation may be limited.	Projects may not reflect community values and needs.
Partner ^3^	Smaller retailers that serve an outsized role in communities with limited food access may find it challenging to participate because of complexities in processing incentives; procuring, storing, and displaying FVs; and marketing to SNAP participants.	GusNIP may not be accessible to certain retailers or available where SNAP participants prefer to shop.
Individual ^4^	Current funding levels allow issuance of incentives to only a small fraction of SNAP participants.	Most SNAP participants cannot access GusNIP.
	People with low incomes who are ineligible for SNAP cannot receive incentives.	People experiencing food and nutrition insecurity but not meeting SNAP eligibility guidelines cannot benefit from GusNIP.
	Program features such as caps on monthly benefits or requirements for participants to match incentives with their limited SNAP funds may create barriers to participation.	SNAP participants with access to GusNIP may still not be able to participate.

^1^ Community—a geographic site in which a GusNIP program operates. ^2^ Organization—an organization that seeks GusNIP funding and/or implements GusNIP programs in its community. ^3^ Partner—an organization or business entity (including food retailers and local farmers) that partners with an organization implementing a GusNIP program. ^4^ Individual—a person who receives GusNIP benefits.

**Table 4:** Opportunities to advance equity in GusNIP in the farm bill.

Opportunity	Recommendations	Impact on Equity
1. Increase total GusNIP funding	Increase GusNIP appropriations overall to $7 billion over 10 years.	Expand program reach to a larger proportion of SNAP participants and fund additional budget additions/modifications to advance equity for impacted communities.
2. Increase funding and support to lower-resourced organizations and impacted communities	Increase funding for pilot projects.	Allow lower-resourced organizations in impacted communities to develop the experience and skills to implement subsequent larger projects, while not competing with higher-resourced organizations applying for large-scale projects.
	Increase funding to the GusNIP Nutrition Incentive Program Training, Technical Assistance, Evaluation and Information Center (NTAE).	Increase availability and scope of technical assistance GusNIP NTAE provides to better support lower-resourced organizations and impacted communities to secure GusNIP grants and implement projects.
	Expand provisions of planning grants that allow prospective applicants to engage in community-based application development, needs assessment, and project planning.	Offer lower-resourced organizations and impacted communities resources and technical support to engage community members in the planning process and prepare strong applications centering community-identified needs for GusNIP project funding.
3. Eliminate the match requirement	Eliminate the requirement for grantee-generated matching funds.	Reduce a financial barrier to GusNIP participation that disproportionately impacts lower-resourced organizations and impacted communities.
	If sufficient funds are not available to eliminate entirely the matching requirement, we recommend the following intermediate steps, in order of preference:	
	Limit the proportion of costs that must be covered by grantee-generated matching funds to no more than 10%.	
	Replace a universal match requirement with a more flexible one that reduces the size of the match requirement for lower-resourced organizations and impacted communities.	
	Reduce the portion of grant funds requiring match (for example, only program costs should require matching funds, not NIs) and expand what qualifies as matching funds.	
4. Support statewide expansion	Allocate funds to support statewide SNAP incentive expansion including state administration, EBT integration, and centralized EBT payment processing.	Fund critical intermediate step towards national expansion and universal access to NIs for all SNAP participants.
5. Expand and diversify retailer participation	Provide community-owned food retailers with technical and financial support for implementing electronic NI issuance and redemption technology.	Eliminate time and resource barriers to GusNIP participation for small and independent retailers that may play an outsized role as retailers for SNAP participants in impacted communities.
6. Expand program marketing	Develop federally supported, national GusNIP promotions and offer supplemental funding to grantees for community-level promotional activities.	Increase awareness and uptake of existing GusNIP programs through media and messaging that is culturally and linguistically tailored to diverse audiences.

## Data Availability

No new data were created beyond what are shared in the manuscript.

## Supplementary Materials

The following supporting information can be downloaded at: https://www.mdpi.com/article/10.3390/nu15234863/s1, Table S1: Opportunities to advance equity in GusNIP through USDA administrative action.
