# Supplementary material for: Advancing Equity in the Farm Bill: Opportunities for the Gus Schumacher Nutrition Incentive Program (GusNIP)
Source: Nutrients. 2023 Nov 21;15(23):4863. doi: 10.3390/nu15234863 (PMC10707921; doi:10.3390/nu15234863)
Supplement: Supplementary file 1 [file nutrients-15-04863-s001.zip › nutrients-2641392-supplementary.pdf]

Table S1. Opportunities to advance equity in GusNIP through USDA administrative action.

Opportunity	Recommendations	Impact on equity
Increase funding and support to lower-resourced organizations and impacted communities	Award a minimum number of grants or a minimum proportion of funds to lower-resourced organizations or impacted communities. This could be accomplished from a pool of grant funds awarded through a single request for application (RFA), or by issuing a separate RFA with a dedicated funding level for which only lower-resourced organizations or impacted communities are eligible.	Increase likelihood of funding projects in impacted communities led by lower-resourced organizations.
	Engage and support the NTAE to (a) develop criteria to define lower-resourced organizations and impacted communities, and (b) help potential grantees determine whether their organization or community qualifies for such designation	Identify lower-resourced organizations and impacted communities that would benefit from GusNIP funding.
	Modify grant application scoring so that meeting the following criteria increases the likelihood of funding:<br>• How well the proposed project addresses diet quality and diet-related disease disparities.<br>• How data and other strategies are used to identify, reach, and engage impacted communities.<br>• How well applicant leadership reflects the impacted community and/or is connected with the impacted community as evidenced by experience working in or relationships with the community.<br>• How applicant will share power with the impacted community and develop equitable participation and leadership opportunities for community members.<br>• The extent to which minority- and women-owned businesses are included as retailers.	Increase likelihood that GusNIP grants support projects that address disparities, involve people from, impacted communities in leadership roles, share power with impacted communities, and include minority- and women-owned businesses as retailers.
Increase community participation and leadership in GusNIP	Require applicants to demonstrate they authentically incorporate the values and needs of their communities into their projects. Applicants must partner with community organizations and engage with community members to ensure community needs are identified and prioritized	Increase likelihood that GusNIP grants support projects that genuinely address impacted community concerns and respect community values involve people from impacted communities in

	throughout program development, implementation, and evaluation. A coalition or collaborative that includes community partners in leadership roles is one structure that can facilitate this.<br><br>Require applicants to create equitable decision-making structures and processes that lead to power-sharing with communities and to describe plans to share information with the community (including how grant funds are used and who receives them, and project impacts and who is benefitting from them). At a minimum, each project should have a Community Advisory Board.<br>Require applicants to use a community-driven needs assessment process. Applicants needing support for compiling existing assessment data or conducting an assessment could apply for a planning grant and/or receive data and technical support from the NTAE.<br><br>Include community-based organizations and people with lived experience from impacted communities in review of grant applications.<br><br>Require data in grantee reports on roles played by community members during project implementation.<br><br>Require a minimum proportion of grant funds be subcontracted to community-based organizations whose boards, leaders and staff reflect the community being served.<br><br>Require grantees to pay community members for their involvement, e.g., as project staff salaries or for time and expenses spent serving as Community Advisory Board members.	leadership roles, and share power with impacted communities.
Reduce burden of the match requirement	Provide technical assistance to lower-resourced organizations and impacted communities to help them generate matching funds.	Increase likelihood that less-resourced organizations can meet match requirements.

	Clarify and expand what grantee contributions are eligible for inclusion as in-kind contributions.	
Support statewide expansion	Provide technical and financial support for implementing electronic nutrition incentive issuance and redemption technology to farmers' markets and community-owned food retailers that serve impacted communities.<br><br>Support partnerships between state agencies and non-profits to upgrade EBT technology to integrate nutrition incentive programs and SNAP.<br><br>Work with companies (e.g., FIS, Conduit) to create less costly, standardized, centralized options for integrating incentives into EBT processing technology for use by all states.	Reduce barriers faced by less-resourced organizations and retailers to participating in electronic nutrition incentive issuance and redemption.
Expand and diversify retailer participation	Consider the number and type of community-owned and rural retailers that have agreed to participate in the GusNIP project when scoring GusNIP grant proposals.<br><br>Assure grantees have the capacity (time, skills, and resources) to provide technical assistance to community-owned retailers for setting up POS systems (or for transitioning to use of the SNAP EBT system for incentive redemptions). NTAE could support grantees in developing these skills and resources given adequate funding<br><br>Support grantees in engaging local vendors to supply FVs to GusNIP retailers, not only in farmer's markets but also in bodegas, corner stores, and other smaller stores<br><br>Expand other types of support for community-owned businesses participating in GusNIP through loans, expanded Small Business Administration technical support, and procurement preferences.<br><br>Include support for retailers to integrate nutrition incentives in the SNAP EBT Modernization	Increase participation by community-based, minority-owned, and rural retailers.

	Technical Assistance Center, as online SNAP becomes more widely and equitably available	
Expand program marketing	Develop federally-supported, national GusNIP promotions to grantees for community-level promotional activities.<br>Include participation by community partners in place-based project outreach and marketing.<br><br>Require GusNIP applicants to explain their approach to reaching diverse potential participants, such as those with language barriers, and to budget sufficient award funds to support the proposed approach.<br><br>Encourage NTAE to create resources describing effective and scalable evidence-based marketing strategies.<br><br>Increase USDA promotion of the Shop Simple with MyPlate app and its GusNIP retailer and program locator function.<br><br>Encourage use of SNAP-Ed funds for social marketing strategies to increase SNAP-Ed reach and GusNIP awareness, such as digital advertising on social media and apps. SNAP-Ed funds should support outreach, marketing and engagement activities conducted by community partners.	Assure that people from impacted communities or otherwise marginalized populations are aware of GusNIP programs and participate in them.
